# Editorial: Novel strategies to repair the infarcted heart, volume II

**DOI:** 10.3389/fcvm.2024.1379240

**Published:** 2024-03-07

**Authors:** Anke M. Smits, Sveva Bollini, Monika M. Gladka

**Affiliations:** ^1^ Department of Cell and Chemical Biology, Leiden University Medical Center, Leiden, Netherlands; ^2^ Department of Experimental Medicine (DIMES), University of Genova, Genova, Italy; ^3^ Cellular Oncology Unit, IRCCS Ospedale Policlinico San Martino, Genova, Italy; ^4^ Department of Medical Biology, Amsterdam Cardiovascular Sciences, Amsterdam University Medical Centers, Amsterdam, Netherlands

**Keywords:** cardiac repair, regeneration, cardioprotection, novel treatments, cardiac ischemia and injury

**Editorial on the Research Topic** Novel strategies to repair the infarcted heart, volume II

Due to the remarkable success of our research topic titled “Novel Strategies for Healing the Infarcted Heart”, we are delighted to announce the launch of Volume II, featuring an expanded collection of articles.

In this Research Topic, Rolland and Jopling provide a comprehensive outline of cardiac regeneration. The structural and functional reconstitution of the injured heart still represents a significant clinical need towards which translational research has dedicated increasing effort over the years. The first evidence of endogenous mechanisms of bona fide cardiac regeneration, based on the sustained myocardial renewal response observed in lower vertebrates (i.e., zebrafish) and early mammalian neonates after myocardial infarction (MI), has ignited further studies to pinpoint new molecular targets for future therapy.

In their review, the authors offer an accurate overview of the most informative mammalian and non-mammalian preclinical models used to investigate endogenous cardiac regeneration, as well as a critical discussion on the different processes and the underlying heart restoration. While proficient reactivation of cardiomyocyte proliferation indeed represents a primary focus of the investigation, Rolland and Jopling also address in detail the crucial role of the multicellular interplay within the cardiac microenvironment, significantly contributing to the regeneration process. Indeed, bona fide cardiac regeneration is a multifaceted process based on the functional integration of different components, overall modulating cardiomyocyte behavior. These may include immune response cells associated with cardiac injury, endothelial cells and lymphatic vasculature, epicardial stromal cells, fibroblasts and extracellular matrix components.

Another relevant and recent line of investigation presented in this Research Topic focuses on the influence of mechanical forces and mechano-transduction on myocardial renewal. As a matter of fact, in their original research article, Rolland et al. suggest that mechanical loading alteration due to cardiac injury may sustain cardiac regeneration in adult zebrafish.

Combining *in vivo* zebrafish models and molecular techniques, they identified a new therapeutic target on cardiomyocytes in the mechanosensitive ion channel *Trpc6a*. *Trpc6a* acts as a mechanosensor regulating the AP1 transcription complex, which has been recently described as acting with a dual mechanism in non-mammalian vs. mammalian regenerative models. While in adult zebrafish, AP-1 transcription factors have been previously shown to influence the chromatin landscape, allowing activation of gene expression programs leading to cardiomyocyte proliferation (1), an independent study on rodent post-natal cardiomyocytes has highlighted the role of the AP-1 complex as a functional regulator promoting cardiomyocyte maturation over proliferation (2). This evidence suggest that further investigation on mammalian vs. non-mammalian cardiac regenerative models is required in order to better understand the molecular mechanisms driving the transition from full restoration into defective repair during species evolution.

Many forms of cardiovascular disease, including myocardial infarction, are associated with cardiac fibrosis, which negatively impacts the progression of the disease. Therefore, anti-fibrotic therapy may be an appealing approach to enhance cardiac function after injury. The main cellular actors in the fibrotic process are the activated cardiac fibroblasts (myofibroblasts) that can derive from different cell types. Although the activation process is quite well understood, the identification of therapeutic targets has been scarce. In their original research publication, Moita et al., argue that this is partly due to a lack of reliable cell culture models for fibroblasts. To address this question, the authors provide extensive transcriptomic and proteomic data on quiescent and activated cardiac fibroblasts (CF) from different sources under defined conditions. Their analysis reveals several potential cardiac fibrosis targets that were not explored in the heart but have been investigated in other fibrotic or oncological settings, suggesting the cardiac field may benefit from leveraging insights from oncologic programs.

The road to cardiac repair covers many different types of strategies, each with its own challenges on the way to clinical application. The review by Sun et al., provides insight into the current approaches within the cardiac regenerative field, ranging from the early days of direct cell transplantation to paracrine approaches such as growth factors and exosomes. Relatively new applications in cardiac repair are discussed, including the use of biomaterials to either increase retention of cells or drugs or to provide support to the injured heart. The authors provide an insightful overview of (cardiac) biomaterials and their applications, especially for researchers not so familiar with this subject. Overall, this review demonstrates the progress in the field of cardiac repair, but also concludes that designing of a long-acting, safe and effective treatment that is acceptable to both doctors and patients is still a long way to go.

The onset of a MI is a multifaceted process characterized by the involvement of numerous immune cells, cytokines, chemokines, and other circulating effector proteins. Emerging evidence highlights the pivotal role of these components in myocardial remodeling and fibroblast activation at the infarct site (3). The study conducted by Doudin et al., focused on the impact of cooperative DNA binding involving the cytokine-driven transcription factor STAT1 in a murine model of acute MI. The authors utilized STAT1 knock-in mice carrying a specific genetic mutation (F77A), known for its capability to disrupt STAT1 tetramerization and cooperative DNA binding. Notably, the presence of the F77A mutation in STAT1 seemed to have a positive influence on the survival rates of male mice while simultaneously mitigating left ventricular dysfunction in female mice following MI. This outcome correlated with a shift in the STAT3/STAT1 protein ratio within the knock-in mice, which, in turn, led to heightened immune cell infiltration and an upregulation of genes related to inflammation. Furthermore, the authors observed a more pronounced downregulation of genes associated with oxidative phosphorylation and metabolic pathways in the knock-in mice. Overall, the authors show that lack of STAT1 cooperative DNA binding stimulates early infiltration of immune cells into the infarct area and protects against adverse cardiac remodeling. Yet, the molecular mechanisms underlying improved hemodynamic outcomes and functional recovery in ischemic hearts still need to be defined.

Within this research topic, we reiterate that regeneration is a complex process encompassing various facets. This involves the precise regulation of intrinsic cardiac processes, encompassing cardiomyocyte proliferation, inflammation, fibrotic response, and the impact of mechanotransduction and the microenvironment. It is critical to consider all these factors when exploring novel and enhanced therapeutic strategies.
